# Imaging of prostate micro-architecture using three-dimensional wide-field optical coherence tomography

**DOI:** 10.1364/BOE.537783

**Published:** 2024-11-14

**Authors:** Marta K. Skrok, Szymon Tamborski, Matt S. Hepburn, Qi Fang, Mateusz Maniewski, Marek Zdrenka, Maciej Szkulmowski, Adam Kowalewski, Łukasz Szylberg, Brendan F. Kennedy

**Affiliations:** 1 Institute of Physics, Faculty of Physics, Astronomy and Informatics, Nicolaus Copernicus University in Toruń, 5 Grudziądzka St., 87-100 Toruń, Poland; 2 Department of Electrical, Electronic and Computer Engineering, School of Engineering, The University of Western Australia, 35 Stirling Highway, Perth 6009, Western Australia, Australia; 3 BRITElab, Harry Perkins Institute of Medical Research, QEII Medical Centre Nedlands and Centre for Medical Research, The University of Western Australia, Perth, Western Australia 6009, Australia; 4 Department of Obstetrics, Gynaecology and Oncology, Chair of Pathomorphology and Clinical Placentology, Collegium Medicum Jan Biziel University Hospital, 75 Ujejskiego St., Bydgoszcz 85-168, Poland; 5 Department of Tumor Pathology and Pathomorphology, Oncology Centre, Prof Franciszek Łukaszczyk Memorial Hospital, 2 Romanowskiej St., Bydgoszcz 85-796, Poland; 6 Center of Medical Sciences, University of Science and Technology, 7 Kaliskiego St., Bydgoszcz 85-796, Poland

## Abstract

Prostate cancer is a global health issue that requires new diagnostic methods to provide accurate and precise visualization of prostate tissue on the micro-scale. Such methods have the potential to improve nerve-sparing surgery and to provide image guidance during prostate biopsy. In this feasibility study, we assess the potential of *en face* three-dimensional wide-field optical coherence tomography (OCT), covering a volumetric imaging field-of-view up to 46 × 46 × 1 mm^3^, to visualize micro-architecture in 18 freshly excised human prostate specimens. In each case, validation of contrast in OCT images is provided by co-registered wide-field histology images. Using this co-registration, we demonstrate that OCT can distinguish between healthy and cancerous glands at different stages, as well as visualize micro-architecture in the prostate, such as epineurium and perineurium in nerves and the tunica intima and tunica media in blood vessels.

## 1. Introduction

Prostate cancer is the second most frequently diagnosed cancer in men and represents 7% of all new cancer cases worldwide [1]. Medical imaging plays a critical role in the treatment of prostate cancer with the primary aim of detecting cancer early and precisely [2]. For example, a gold standard in diagnostic imaging of prostate cancer is transrectal ultrasonography (TRUS) [3]. In clinical practice, other methods are also used, particularly computed tomography, magnetic resonance imaging, and positron emission tomography [4]. However, clinical practice still lacks intraoperative imaging tools capable of visualizing prostatic structures on the micro-scale. Such imaging capabilities are needed in several aspects of prostate cancer treatment. For example, during biopsy guidance, TRUS is often used to provide image-guided biopsy, however, the relatively low spatial resolution of TRUS contributes to >30% of TRUS-guided biopsies providing false negative results [5]. Another challenge in prostate surgery is performing nerve-sparing surgery accurately. Current radical prostatectomy methods cause erectile dysfunction in >70% of patients [6]. Nerve-sparing prostatectomies are performed but the challenge of visualizing nerves and cancer during surgery results in positive margins in up to 30% of cases [7,8]. Therefore, there is a pressing need to develop intraoperative methods enabling surgeons to better visualize nerves and to differentiate between benign and malignant tissues. To address this, a range of optical methods have been proposed for intraoperative prostate imaging, including optoacoustic techniques [9,10], Raman spectroscopy [11,12], confocal fluorescence microscopy [13,14], light-sheet microscopy [15,16], and optical coherence tomography (OCT) [17,18]. Of these methods, OCT holds particular promise, as it can be performed on fresh tissue and provides a combination of rapid volumetric acquisition (readily covering several millimeters in less than a second), micro-scale spatial resolution (∼5-10 µm), and imaging depths up to 1 mm in dense tissue. Furthermore, it is straightforward to implement compact OCT imaging probes, an important consideration for eventual clinical translation. OCT has been proposed both as a method for the detection of prostate cancer [19] and to improve nerve-sparing surgery [20].

An early study using time domain (TD) OCT on seven fresh biopsy specimens demonstrated a contrast between different prostate micro-structures, validated by histology [21]. However, the image quality was relatively low compared to modern OCT systems and only two-dimensional (2-D) images (B-scans) were acquired. Since then, studies have been performed using full-field OCT on biopsy specimens obtained from eight patients [22] and OCT attenuation imaging on a single prostate [23]. These studies demonstrated that OCT provides visualization of a range of prostate micro-structures, including healthy and cancerous glands, fibromuscular stroma, blood vessels, and nerves. However, these studies were performed on fixed tissue, which is known to significantly alter OCT image contrast [24], making it difficult to assess its potential as an intraoperative tool. Another study, conducted on 12 fresh prostate specimens, showed that µOCT can visualize prostatic structures, such as benign and cancerous prostate glands, as well as cancer-related crystalloids, and corpora amylacea [25]. While this technique offers high spatial resolution (1 µm), it is limited to imaging transverse fields-of-view only up to ∼1.0 mm^2^, posing a challenge for its clinical translation [25]. Also, whilst the high spatial resolution demonstrated in this study improves visualization of the prostate, it is challenging to achieve such resolution in imaging probes that will eventually be needed for intraoperative use.

Several studies have also focused on the development of needle-based OCT to improve the accuracy of image-guided biopsy [26,27]. An *ex vivo* study on 20 prostate specimens demonstrated the potential to distinguish healthy tissue from malignant tissue [26]. A preliminary *in vivo* study has also been performed on two patients, demonstrating safety and clinical feasibility [27].

An early *in vivo* study on 24 patients, performed using a TD-OCT system, showed the potential of OCT to detect the neurovascular bundle during laparoscopic radical prostatectomy [20]. Another early *ex vivo* study conducted on five specimens, also using a TD-OCT system, showed that OCT can visualize the cavernous nerve [28].

Previous OCT studies on prostate tissue have demonstrated that it has the potential to improve both the diagnosis and treatment of prostate cancer. However, there remains of paucity of studies that show the extent to which OCT can be used for 3D assessment of the micro-architecture of the prostate in fresh tissue, making it challenging to assess the potential of OCT for intraoperative use. In addition, a more comprehensive study of OCT on freshly excised prostate, validated by histology, would provide a valuable reference point against which to benchmark image quality achieved using compact imaging probes, such as needle probes.

In this feasibility study, we performed wide-field 3-D OCT imaging (46 × 46 × 1 mm^3^) on fresh cross-sections of prostate specimens and validated the image contrast by co-registering wide-field *en face* OCT images with wide-field histology. To perform histology, the prostate cross-section was placed in a macro-cassette. Importantly, the use of macro-cassettes allowed the entire prostate cross-section to appear in one histology image, rather than over several histology images, which would be required using standard micro-cassettes. This approach greatly facilitated accurate co-registration between wide-field *en face* OCT images and histology. In total, using this approach, we scanned 18 fresh prostate cross-sections obtained from 11 patients. We demonstrate the potential of OCT to visualize a range of prostate micro-architectures, including healthy and cancerous prostate nerves, blood vessels, and glands. Importantly, we also demonstrate that 3D-OCT can provide contrast of features within these structures, including the epineurium and perineurium in nerves and the tunica intima and tunica media in blood vessels. We also demonstrate that OCT has the potential to distinguish between healthy and cancerous glands and show how OCT image contrast changes at different stages of prostate cancer. Our results also suggest some limitations of OCT imaging of the prostate. For example, in some instances, OCT contrast of healthy glands is reduced by prostatic fluid present within the glands, which, effectively, acts to reduce the refractive index difference between glands and surrounding tissues. Also, whilst OCT can visualize structures within nerves, the contrast of some structures, such as fascicles, is quite subtle. As OCT continues to be developed for use in treating prostate cancer, we believe that our study assists in assessing its clinical potential.

## 2. Methods

### 2.1 Experimental setup

The imaging system is a spectral-domain OCT system based on the Telesto II platform (TEL220C1, Thorlabs) and has been described in detail previously [29]. Briefly, the light source is a superluminescent diode with a central wavelength of 1300 nm and a bandwidth of 200 nm that provides a full width at half maximum (FWHM) axial resolution (in air) of 5.5 µm. The scanning unit comprises a pair of galvanometric scanning mirrors to deflect the beam in both lateral directions and is combined with a scanning lens (LSM04, Thorlabs) providing a FWHM lateral resolution of 13 µm. The working distance is 42.3 mm, which provides sufficient space for both a glass imaging window (6 mm thick, Edmund Optics) between the lens and the sample and to conveniently access the sample. The imaging window was used for two main reasons. Firstly, the system comprises an interferometer in a common-path configuration to provide stable operation in a clinical environment. In this case, the reference beam comes from the same optical path as the light backscattered from the sample. The interface between the window and the sample provides this reference reflection. Secondly, as the fresh prostate specimens had a surface with roughness varying over several millimeters, it was important to flatten the tissue to enable each region of the tissue to be scanned using OCT. This was achieved by compressing the sample against the window.

The scanning head is oriented in standard epi-illumination mode with the imaging beam incident from above the horizontally positioned sample. The imaging system also features a visible light channel decoupled from the object arm of the interferometer using a dichroic mirror with a camera providing a live 2-D preview of the field-of-view of the OCT system to facilitate sample positioning. This task is realized using a motorized 3-axis stage. The spectrometer comprises a line-scan camera with 2,048 pixels, providing an axial imaging range of 3.5 mm. Due to the attenuation of OCT intensity in tissue, the effective imaging depth in the prostate was ∼1 mm. The interference spectra corresponding to axial depth scans (A-scans) are acquired in an exposure time of 12 µs within a line period of 16 µs. We used the following scanning protocol: 808 A-scans per each B-scan, and 2,424 B-scans per *y*-axis. In post-processing, every three B-scan locations are averaged to alleviate the effect of optical noise whilst still achieving isotropic sampling in the *x* and *y* directions. This protocol was repeated in each position twice: with and without mechanical loading from a piezoelectric actuator in which the imaging window was mounted. This was used to enable simultaneous acquisition of optical coherence elastography (OCE) scans [30], which is required in a parallel project to measure the mechanical properties of prostate tissue. The data from these two acquisitions was also averaged to further improve image quality.

**Fig. 1.**
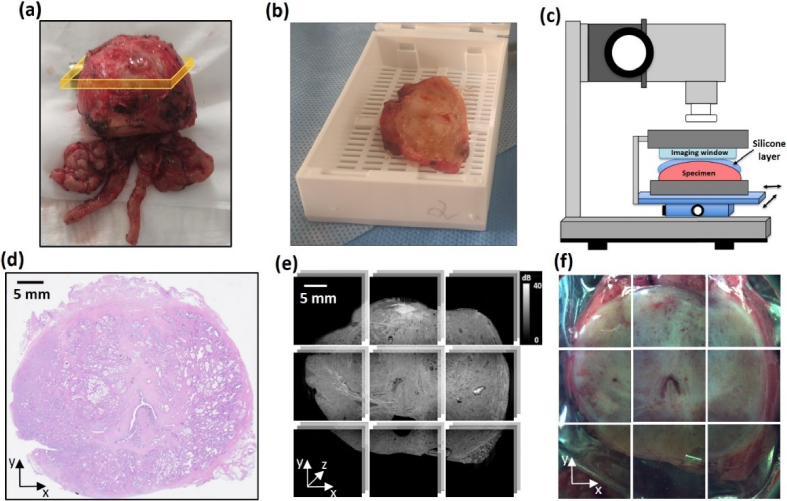
Workflow for OCT acquisition and co-registration with histology. (a) Prostate immediately after radical prostatectomy. The orange band indicates the cross-sectional specimen dissected for OCT imaging. (b) ∼5 mm thick tissue slice obtained from the cross-section, ∼1 cm below the prostate apex. (c) Schematic diagram of wide-field OCT scanning using a translation stage at the bottom surface of the slice. (d) wide-field H&E histology image. (e) Mosaicked wide-field 3D-OCT image obtained by volume stitching. (f) Mosaicked wide-field 2-D camera image of the sample covered with the silicone layer and pressed against the imaging window during OCT imaging obtained using the visible range camera.

The field-of-view of the imaging system is 16 × 16 mm^2^. To cover the entire surface area of a prostate slice, we used lateral motorized stages and volumetric acquisition in each locations of a 3 × 3 grid with a 1 mm margin overlap of adjacent volumes, following a protocol used previously [29]. Each of the 16 × 16 sub-volumes was acquired in 63 seconds, corresponding to 11 minutes for an entire mosaicked image. *En face* OCT images for a given distance from the sample surface *z* were obtained by averaging consecutive B-scans within the scanning depth range from (*z*-20) µm to (*z* + 20) µm. Using a mosaicking algorithm, wide-field OCT images of 46 × 46 × 1 mm^3^ were generated (Fig. 1(e)). The corresponding 2-D mosaic from a visible range camera was also generated (Fig. 1(f)) and is helpful in co-registering OCT images with histology (Fig. 1(d)). The data acquisition is controlled by custom software.

### 2.2 Clinical scanning

The samples were prepared by qualified pathologists. In each case, after radical prostatectomy, the prostate was cut in the cross-sectional plane perpendicular to the apex (Fig. 1(a)), ∼1 cm below the tip of the apex, and was then transported to the OCT imaging system. In some cases, the slice was larger than the field-of-view of the wide-field OCT image and was bisected in the lateral plane prior to imaging (N = 5). OCT was performed on the surface that was subsequently prepared for histology. Prior to imaging, the samples were irrigated with 0.9% saline solution and covered with a silicone layer (Elastosil P7676, Wacker Chemie AG, thickness ∼0.5 mm), used to acquire preliminary OCE scans as part of a parallel project. A motorized *z*-axis stage was used to bring the sample into contact with the imaging window (Fig. 1(c)), which flattened the imaged surface and removed residual air bubbles. After scanning, the sample was placed in a macro-cassette with the surface imaged with OCT facing downwards, as this is the surface from which histology images were subsequently generated. An advantage of this approach is that one histology image could be generated from an entire tissue cross-section covering a field-of-view of 45 × 60 mm^2^. This greatly facilitated accurate co-registration, as the use of standard micro-cassettes would require multiple histology images for each cross-section, making it more challenging to correspond features between histology and OCT images. The standard protocol used in the pathology department for the preparation of hematoxylin and eosin (H&E) stained histology images was performed (Fig. 1(d)). In total, 18 samples from 11 prostates were scanned. Each of the samples imaged was subsequently subjected to the standard protocol carried out at the hospital, therefore our ethics did not permit variations to this protocol, so as not to affect the patient's treatment process. The project ethics were approved by the Bioethical Committee of the Oncology Centre in Bydgoszcz.

### 2.3 Co-registration

OCT image contrast was validated through co-registration with gold standard H&E histology. Co-registration, in this case, involved imaging the sample in the same plane as closely as possible with both OCT, on the fresh tissue, and H&E histology, on the fixed sample. This approach enables close co-registration and provides sufficient accuracy for our research as we can clearly see the same features between both OCT and histology. Whilst it was outside the scope of our study, co-registration could be further improved using numerical methods for matching both images. However, this would require precise consideration of nonlinear geometric distortions of section that occur during histological processing of the sample and vary for different tissue types [31]. In particular, fixation of the tissue in formalin causes it to shrink and warp relative to the OCT image of the fresh tissue. However, in most cases, it was possible to find a depth in OCT volumes for which the *en face* image and corresponding histology image represented approximately the same plane. This was determined by co-locating features in the two images. Wide-field images from the visible range camera were also used to assist in identifying the *en face* OCT image that provided the best correspondence with histology. The histology images were then annotated by pathologists, allowing contrast in OCT images to be validated. In the images presented in Figs. 3–7, our goal was to co-register specific features within the prostate specimens. In some instances, this resulted in scale bars of different lengths. We believe that this is mainly caused by the well-known tissue shrinking and warping caused by the histology preparation process. We found that this effect particularly affects nerves, as is visible in Fig. 3, where the size of nerves in OCT images can be up to twice that of corresponding nerves in histology images.

## 3. Results

**Fig. 2.**
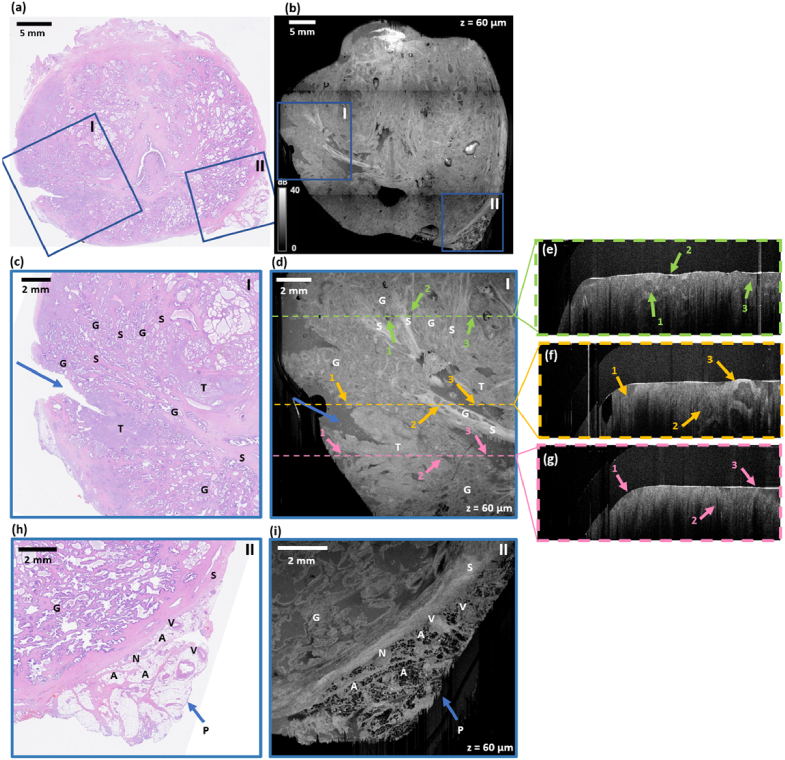
Example of co-registration of (a) wide-field histology and (b) wide-field *en face* OCT of a prostate slice. (c) and (d) present magnified images of the region indicated by the blue box labeled I in (a) and (b), respectively, (e)-(g) show B-scans that correspond to the locations on the *en face* image shown in (d) with characteristic details visible both the B-scans and *en face* images highlighted, and (h) and (i) are magnified images of the region indicated by the blue box labeled II in (a) and (b), respectively. G = healthy gland, T = tumor, S = stroma, P = periprostatic tissue, A = adipose tissue, V = blood vessel, N = nerve. The numbers indicate the corresponding feature in the *en face* OCT images and B-scans.

Figure 2 presents an example of the co-registration achieved between histology (Fig. 2(a)) and wide-field *en face* OCT (Fig. 2(b)) for a representative prostate specimen, where the OCT image in Fig. 2(b) is from a depth of 60 µm. *En face* OCT images obtained for this sample at each scanning depth are shown in Supplementary Visualization 1. The blue boxes in Fig. 2(a) and Fig. 2(b) highlight examples of corresponding regions in both images. Magnified images of these regions are presented in Figs. 2(c),(d),(h),(i).

As can be seen in Figs. 2(c)–2(d), a main factor that can be used to confirm co-registration between histology and OCT images is correspondence of the tissue boundaries between both images. The arrows in Fig. 2(c) and Fig. 2(d) highlight a characteristic tear on the periphery of the specimen that is visible in both images. Image co-registration can also be demonstrated by analyzing the morphology of individual image features. In Fig. 2(c) and Fig. 2(d), we can distinguish three main types of tissue: healthy glands (G), tumor (T), and stroma (S). In the OCT image, healthy glands appear as regions of high heterogeneity. On the other hand, cancer, in this case, appears as a region with a relatively homogeneous structure. Stroma presents in the OCT image as a region of high intensity.

Figures 2(e)–2(g) show B-scans corresponding to the locations marked in Fig. 2(d). In each case, characteristic features that are visible both in *en face* OCT images and in B-scans are marked with appropriate colors and numbers. These B-scans show that comprehensive imaging of prostate tissue requires the use of 3-D techniques, because the course of individual structures (e.g. glands, particularly visible in Fig. 2(f)) takes place in a plane oblique to the surface of the imaged sample, so imaging at one depth may be insufficient for a detailed analysis of the properties of the examined specimen.

**Fig. 3.**
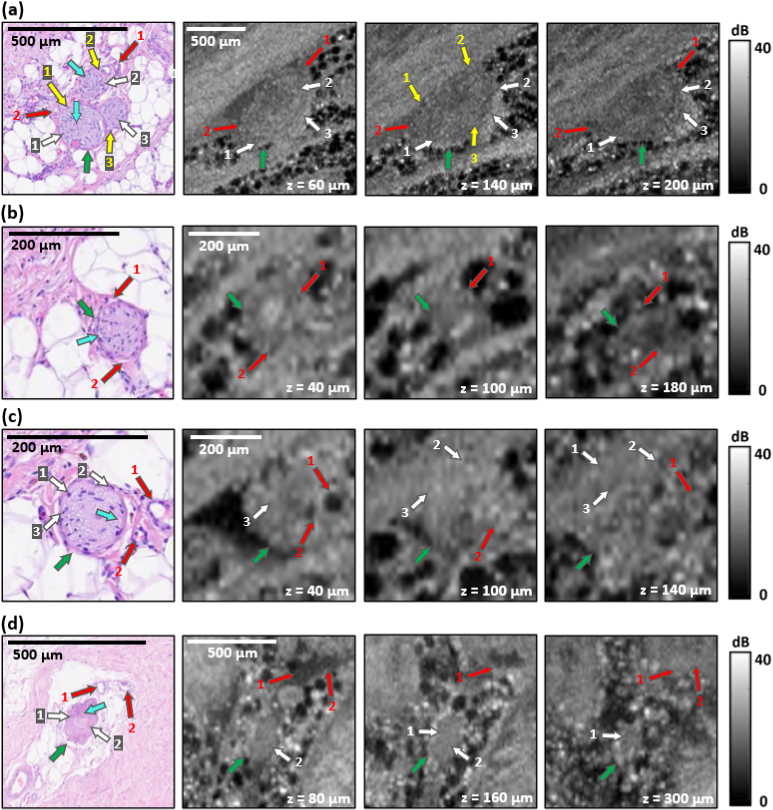
Four examples of histology and *en face* OCT images of nerves present in periprostatic tissue. In each case, the first image presents histology, followed by three co-registered OCT *en face* images from three depths. The depth below the tissue top surface of each image is indicated on the bottom right. The yellow arrows correspond to the perineurium, the blue arrows correspond to axons, the white arrows correspond to axons wrapped in the myelinated structures of Schwann cells, the red arrows correspond to blood vessels, and the green arrows correspond to the epineurium. The numbers indicate the corresponding feature in the histology and OCT images.

**Fig. 4.**
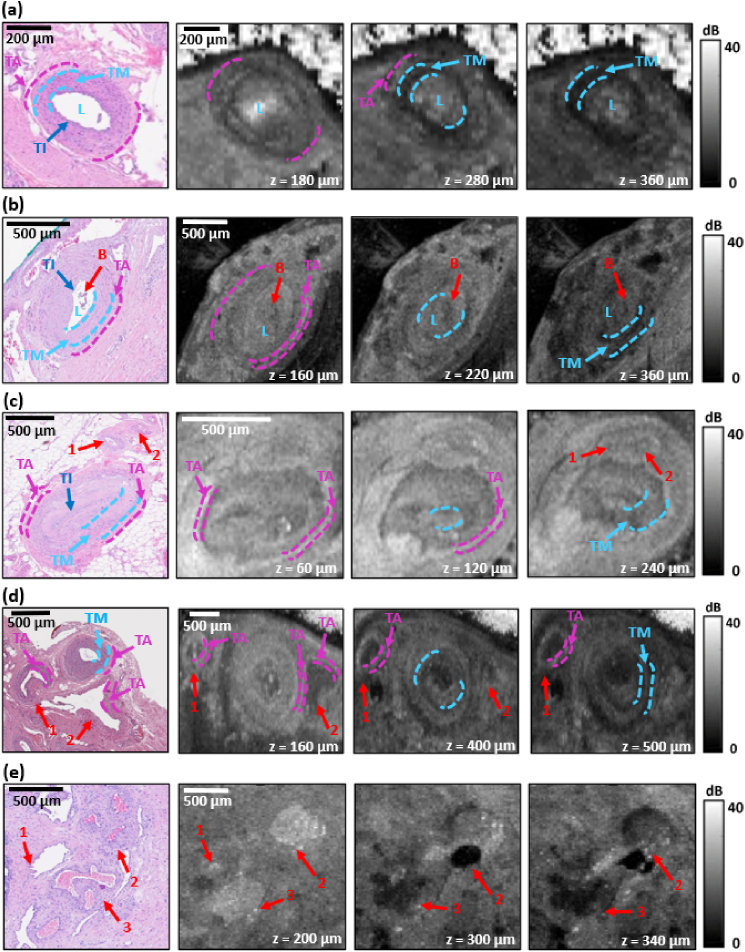
Five examples of histology and *en face* OCT images of blood vessels located within periprostatic tissue. The blue lines mark the boundaries of individual layers in the blood vessel wall: TI = tunica intima, TM = tunica media, TA = tunica adventitia. L = vessel lumen, B = blood within blood vessels. The red arrows indicate the location of small blood vessels. The numbers indicate the corresponding feature in the histology and OCT images.

**Fig. 5.**
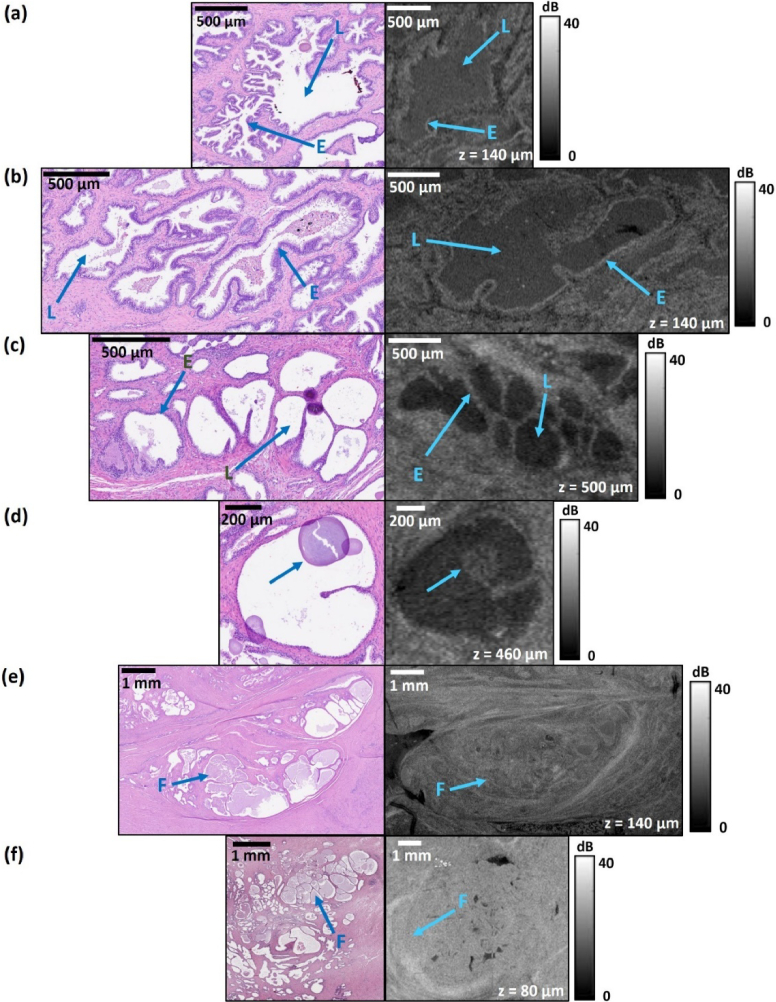
Histology and *en face* OCT images of various types of healthy prostatic glands: (a)-(b) most anatomically typical, (c) atrophic, (d) atrophic with the presence of corpora amylacea, (e)-(f) containing prostatic fluid. E = cellular envelope, L = glands lumen, F = fluid within the healthy gland.

**Fig. 6.**
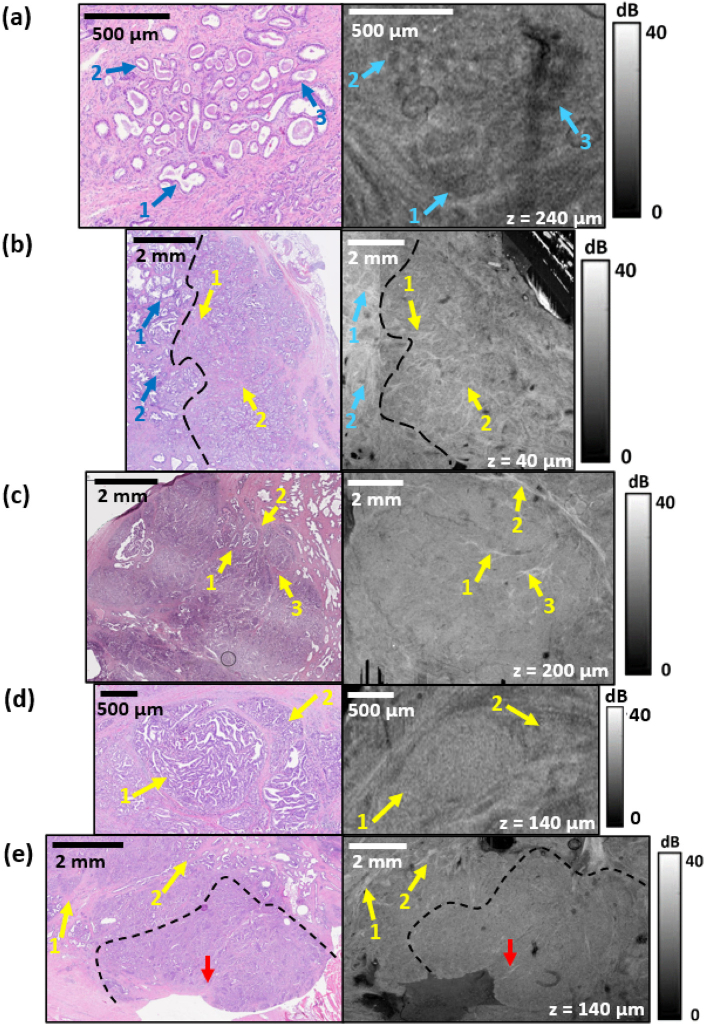
Histology and *en face* OCT images of isolated single areas of tumor tissue classified as (a) Gleason Score 3, (b) Gleason Score 4 (on the right part) and 3 (on the left part), (c)–(d) Gleason Score 4 + 4, and (e) Gleason Score 4 + 5. The blue arrows indicate the distinguishable within Gleason Score 3 area glands, the yellow arrows indicate the characteristic features within classified as a Gleason Score 4 region: bands of connective tissue and cribriform glandular formations, the red arrows indicate the connective tissue within Gleason Score 5 area. The numbers indicate the corresponding feature in the histology and OCT images.

Figures 2(h)–2(i) show another magnified region where co-registration between the histology and OCT image is observed from correspondence the morphology of both images. An area of healthy glands (G) is visible in the top half of the histology image. In the OCT image, this corresponds to a region with clearly visible variations in intensity between the lumen of glands, the cellular envelope of the glands, and surrounding fibrous tissue. Below the regions containing glands, a band of high OCT intensity is visible and corresponds to a region of stroma (S) in the histology image. Adjacent to this region of stroma, a region of periprostatic tissue (P) is visible in the histology image. This tissue is adjacent to the prostate and is of clinical importance because the presence of cancer cells in this region increases the likelihood of cancer metastasis to adjacent organs [32,33]. The presence of periprostatic tissues in the samples scanned in this study depends on the margin excised during prostatectomy, therefore, this region was visible in a subset of the specimens scanned (N = 12). The periprostatic tissue is characterized by prominent regions of adipose tissue (A), which is visible as a honeycomb-like structure in the OCT image, similar to how it presents in OCT images of other tissues, such as breast [34]. The periprostatic tissue also contains other well-defined micro-structures that are visible in the OCT image including blood vessels (V) and nerves (N). In Figs. 3–6, we provide a more detailed description of prominent features visible in OCT images. In each case, co-registration with histology is provided for validation.

Figure 3 presents representative histology and OCT images of nerves within prostate specimens. In each case, *en face* OCT images are presented at three depths to highlight the value of 3D-OCT imaging of the prostate. Importantly, nerves are located within the periprostatic tissues and their visualization is one of the key factors determining the success of nerve-sparing prostatectomy [35,36].

The histology image in Fig. 3(a) presents a neurovascular bundle. The entire nerve bundle is surrounded by the epineurium (green arrow), which is the outer part of the neurovascular bundle. The nerves are arranged into fascicles surrounded by the perineurium (yellow arrows). Within the fascicles, axons (blue arrows) and Schwann cells (white arrows) are indicated by punctuated staining. Several blood vessels (red arrows) are also visible. Figure 3(a) also shows corresponding *en face* OCT images obtained at scanning depths of 60 µm, 140 µm, and 200 µm, respectively. The neurovascular bundle is visible in the OCT images as an area of heterogeneous intensity separated from the surrounding adipose tissue by a thin bright envelope that corresponds to the epineurium (green arrow). The epineurium is visible at each of the presented scanning depths, but the highest contrast is achieved at depths of 60 µm and 140 µm, respectively. At a depth of 140 µm, lighter bands are also subtly visible which likely correspond to the perineurium separating individual fascicles (yellow arrows). Within the nerve bundle, small, circular structures with low OCT intensity are visible in different locations at each depth and likely correspond to axons wrapped in the myelinated structures of Schwann cells (white arrows). Within the neurovascular bundles, slightly larger, dark structures surrounded by a higher-intensity rim are also visible (red arrows). From comparison with histology and considering the diameter of these structures ( ∼50 µm), it is likely that they correspond to blood vessels. Regardless of depth, single axons were not distinguishable on OCT images. Additional examples of nerves are presented in Figs. 3(b)–3(d). In these images, a similar contrast is visible as described for Fig. 3(a).

Figure 4 presents representative histology and OCT images of blood vessels located within the periprostatic region of prostate specimens. As in Fig. 3, in each case, *en face* OCT images are presented at three depths. Figure 4(a) shows a representative artery with its individual layers highlighted. In the central region of the blood vessel, the lumen of the artery (L) is visible. The innermost layer of the artery is the tunica intima (TI), composed of a single layer of endothelial cells and an elastic lamina. In the histology image, it is visible as a thin, clearly stained layer defining the diameter of the artery lumen (dark blue arrow). The middle layer is the tunica media (TM), which comprises muscle and connective tissue, as well as an outer elastic lamina. Histologically, it presents as a darker structure with a visible circular arrangement of smooth muscle fibers (light blue arrow). The outer layer of the artery is the tunica adventitia (TA), which comprises connective tissue and is visible in the histological image as a lighter rim surrounding the whole artery (pink arrows). In the corresponding OCT images in Fig. 4(a), individual anatomical structures of the artery are visible at different scanning depths. At a depth of *z* = 180 µm, the outer perimeter of the blood vessel is visible as a region of lower OCT intensity compared to the surrounding tissue. The internally located artery lumen presents as a region of high OCT intensity, which is probably caused by high optical backscattering from the residual blood within the artery lumen. At a depth of 280 µm, the inner diameter of the artery can be clearly distinguished, visible as a dark ring corresponding to the TM (light blue arrows) which surrounds the bright lumen of the blood vessel. At this depth, the outermost layer of the artery wall, the TA, is also visible and is characterized by high OCT intensity (pink arrows). It should be noted, however, that both the TM and TA are not visible around the entire circumference of the artery at this scanning depth. The TM is most distinguishable along its entire extent as a low-intensity ring at scanning depth *z* = 360 µm. The TI is not visible in the OCT images in Fig. 4, as the single layer of endothelium cells comprising this layer has a thickness of 0.1 to 10 µm [37], which is below the resolution of the OCT system used.

Another example of an artery is presented in Fig. 4(b). As in Fig. 4(a), the layers of the artery wall, *i.e.*, TI, TM, and TA are labelled in the histology image. Additionally, blood (B) remaining after histology processing is also visible within the lumen (L). In the corresponding OCT images in Fig. 4(b), the outer border of the artery with the high OCT intensity TA visible around the entire circumference is most clearly visible at a depth of *z* = 160 µm. The best visualization of the lumen of the vessel (L) was achieved at a depth of *z* = 220 µm, while the low OCT intensity TM was best distinguished at a depth of *z* = 360 µm. At each depth, the blood (B) is visible as a darker area located centrally within the lumen (L).

Figure 4(c) presents a histology image of an artery with adjacent smaller blood vessels, marked with red arrows. The corresponding OCT images show significant variation in the shape of the large artery at different depths. This may be caused by the course of this vessel in the examined prostate slice deviating significantly from the plane perpendicular to the scanning plane. It seems that the course of the adjacent smaller blood vessels (marked with red arrows) is also significantly different from the plane perpendicular to the scanning plane, as they only become visible in the OCT images at a depth of *z* = 240 µm.

Figure 4(d) presents a histology image containing arteries (marked with red arrows), and two veins (marked with blue arrows). In the case of veins, distinct from arteries, the TM is a very thin layer, resulting in the TA typically being the thickest layer within veins. The absence of a thick muscular layer in veins contributes to their compression by adjacent structures, which may result in their appearance in histology images as flattened structures with a shape significantly different from the regular, oval shape of the arteries. In the corresponding OCT images in Fig. 4(d), the veins appear as dark regions, corresponding to the vessel lumen, surrounded by a high OCT intensity ring, corresponding to the TA, the boundaries of which are marked with blue lines. The visibility of the veins in the OCT image, as well as their shape, varies significantly with depth. At depths of *z* = 160 µm and *z* = 400 µm, both veins are visible, although their appearance varies. At a depth of *z* = 500 µm, only one vein is visible.

Another histology image of a region containing veins is presented in Fig. 4(e), with the lumen of two of them, labelled 2 and 3, respectively, almost entirely filled with blood. As shown in the corresponding OCT images in Fig. 4(e), this blood presents as both high and low OCT intensity regions, depending on the depth. For example, at a depth of *z* = 200 µm, both blood-filled veins are visible as regions of high OCT intensity. At a depth of *z* = 300 µm, the vein labelled 2 is characterized by greater OCT intensity, while at a depth of *z* = 340 µm, both veins are visible as regions of low OCT intensity. Interestingly, the vein labelled 1 is distinguishable only at a depth of *z* = 200 µm.

Figure 5 provides an overview of the appearance of healthy prostate glands in OCT images. Figures 5(a) and 5(b) show the most typical anatomy of benign prostate glands. In the histology image, benign prostate glands comprise a distinguishable lumen and are clearly separated from neighboring glands by interstitial tissue. A characteristic feature of healthy glands in histology is the presence of two types of cells: radially arranged luminal cells and encapsulating basal cells, which together constitute the irregular, wavy, well-stained epithelia envelope of glands. In the corresponding OCT images in Figs. 5(a) and 5(b), the lumen of healthy glands appears as a well-defined, dark, irregular area surrounded by a lighter envelope. This high OCT intensity envelope, marked with the letter E, corresponds to the outer layer of healthy prostatic glands, however, it is not possible to distinguish between the basal and secretory cells. Figures 5(a) and 5(b) were obtained at the same scanning depth *z *= 140 µm. However, it is noticeable that the OCT intensity of the central lumen of the gland, marked with the letter L, is different, which may be caused by different volumes of prostatic fluid in both cases. A variation of healthy prostate tissue is atrophic prostate glands, which result from natural age-related processes or previous inflammation. They appear in OCT images as lobular-like structures and are clearly distinguishable from each other, but on the periphery, they take on a more regular, cystic shape (Fig. 5(c)). A common finding inside benign prostatic glands is corpora amylacea (Fig. 5(d)). These concentric structures are formed from clusters of aggregated proteins and appear naturally with age, for example, as a result of inflammation. In the OCT image, the corpora amylacea is visible as an oval structure inside a healthy gland. The main function of the prostate glands is to secrete seminal fluid and the presence of this fluid can be also observed within the lumen of healthy glands, as observed in the histology image in Figs. 5(e) and 5(f). In the corresponding OCT images, the opaque prostatic fluid can behave as a refractive index-matching medium, resulting in similar OCT signal intensity between the lumen and adjacent tissue. This effect can make it challenging to identify individual healthy glands in OCT images.

Figure 6 shows representative images of various stages of prostate cancer present in the specimens scanned in this study. The following subsections in Fig. 6 present *en face* images for different scanning depths, selected individually for each sample in order to obtain the best possible image contrast. In clinical practice, a 5-grade Gleason scale is used to assess the stage of cancer, with Gleason Scores 1–2 describing benign tissues and Gleason Scores 3–5 representing malignant tissues [38]. Pathologists typically determine the stage of cancer by providing the two most extensive Gleason Scores present in histology images. Tumor tissue is classified as Gleason Score 3 when single glands are still distinguishable in the histology image, but infiltrating margins are visible. Gleason Score 4 occurs when adjacent glands overlap, forming clusters in which individual structures cannot be distinguished. The most advanced form of cancer Gleason Score 5, corresponds to a situation when the histology image shows a scattered network of cancer cells, and it is not possible to identify the glandular tissue. In OCT images, cancer-affected regions of the prostate generally appear as regions with a more uniform OCT intensity than in healthy regions, with a gradual reduction of OCT intensity heterogeneity with the cancer advancement stage. This is largely attributed to the reduction in contrast of glands in OCT images as cancer advances. The histology image in Fig. 6(a) corresponds to a region classified as Gleason Score 3 and presents glands (indicated by blue arrows) affected by cancer with the presence of infiltrating margins. In the corresponding OCT image, individual glands (marked with blue arrows) are still distinguishable as dark regions surrounded by areas of higher OCT intensity. However, the walls of individual glands are blurred, making it difficult to clearly separate specific glands from adjacent connective tissue. The histology image in Fig. 6(b) presents a region classified as Gleason Score 3 (on the left part) and Gleason Score 4 (on the right part) separated by a dashed line. In the OCT image, in the area corresponding to the Gleason Score 3, individual glands with blurred boundaries (marked with blue arrows) are still distinguishable. The right side of the OCT image, which corresponds to Gleason Score 4, appears as a region with lower variation in OCT intensity variability with clearly distinguishable bright bands (marked with yellow arrows) corresponding to the connective tissue present between the grouped glands affected by the cancer. Figure 6(c) shows another example of a region of Gleason Score 4, which, also presents as a relatively uniform low-intensity OCT image with clearly distinguishable bands of high OCT intensity originating from connective tissue (marked with yellow arrows). Figure 6(d) shows another manifestation of prostate cancer, classified as Gleason Score 4. The histology image presents cribriform glandular formation (marked with red arrows), which appears as clusters of proliferating glands clearly separated from each other. In the corresponding OCT image, these formations are also distinguishable and are visible as well-delineated, relatively homogeneous areas separated by bands of connective tissue, characterized by higher OCT intensity. In turn, Fig. 6(e) presents a part of the prostate section classified as Gleason Score 4 (in the upper part) and Gleason Score 5 (in the lower part) separated by a dashed line. In both the histology and OCT images, the upper part of the figure, corresponding to Gleason Score 4, shows distinct lighter bands of connective tissue (marked with yellow arrows) present between grouped glands affected by cancer. The lower part of the sample, classified as Gleason Score 5, appears in the histology as an area of relatively regular staining, which in turn is visible in the OCT image as a region of low, uniform intensity. In both images, individual strands of connective tissue (marked with red arrows) are still visible, but they are fewer in number and less distinguishable from adjacent tissues than within the region of Gleason Score 4.

Table 1 summarizes the appearance of individual prostate microstructures in OCT images, including nerves, arteries, veins, healthy glands, and tumors as Gleason Score 3­–5. This table was prepared based on conclusions drawn from the analysis of all n = 18 prostate sections subjected to OCT imaging.

**Table 1.** Characteristic features of the appearance of individual prostate microstructures in OCT images.

Tissue type	Characteristic features on OCT images
Nerve	The neurovascular bundle is visible as an area of heterogeneous OCT intensity surrounded by a thin bright envelope (epineurium). Within the neurovascular bundle, small, circular, structures with low OCT intensity (axons wrapped in the myelinated structures of Schwann cells) and less contrasting lighter bands (perineurium) are visible.
Artery	Two layers of the artery wall are visible: characterized by high OCT intensity in the outermost layer (tunica adventitia) and low OCT intensity in the deeper layer (tunica media). The lumen of vessels not filled with blood is visible as a centrally located darker area.
Vein	Irregular dark regions (vessel lumen) surrounded by a ring of high OCT intensity (tunica adventitia).
Healthy gland	Well-defined, dark, irregular area (lumen of empty glands) surrounded by a lighter envelope (gland cells).
Tumor Gleason 3	Individual glands are still distinguishable as dark regions surrounded by higher OCT-intensity regions. However, the boundaries of individual glands are blurred, making it difficult to clearly define the boundaries between individual glands and the connective tissue surrounding them.
Tumor Gleason 4	Area of relatively uniform intensity except for bright bands of connective tissue or an area with regions alternating high and low OCT intensity (in case of occurrence cribriform glandular formation).
Tumor Gleason 5	Relatively uniform region with low OCT contrast.

## 4. Discussion

Previous studies have shown that OCT can identify micro-architecture in the prostate, such as healthy and cancerous prostate glands [26], adipose tissue [23], blood vessels [22], and neurovascular bundles [28]. However, our study represents the first assessment of 3-D wide-field imaging of the prostate, facilitated by corresponding wide-field histology and, moreover, provides the first detailed description of OCT contrast of micro-structures, such as nerves, blood vessels, and glands, in addition to cancer. Also, we believe that this is the first study to describe all these anatomical micro-structures of the prostate in the same study.

A key feature of our study is the comparison of wide-field *en face* OCT with wide-field histology. In the commonly used procedure to perform histology, the prostate tissue is sliced into 43 × 28 mm^2^ lateral sections and each section is placed into its own micro-cassette. Importantly, a key advantage of the 45 × 45 mm^2^ lateral OCT field of view in this study is that it provides the ability to image the entire prostate cross-section. Therefore, in developing our co-registration protocol, rather than comparing multiple histology images that are unnecessarily acquired over smaller 43 × 28 mm^2^ lateral field of views and then stitched together in post processing, we performed histology by placing entire prostate section into a micro-cassette. Whilst this process can introduce an increased risk of sample damage due to tissue tearing and folding, it reduces geometrical and biomechanical changes from slicing the tissue perpendicular to the scanning plane at intervals across the lateral field of view [39]. These changes can reduce the ability to accurately compare prostate microstructures between the two imaging modalities. Despite a small number (N = 5) of samples in our study being excluded from the subsequent analysis due to poor histology image quality, we believe the co-registration protocol developed here improves the accuracy of comparing prostate structures between OCT and histology by better preserving the features of the tissue. It is important to note, as best highlighted in Fig. 2, that it would be challenging to achieve an exact one-to-one co-registration, as the histology process introduces geometrical distortions to the tissue. One possibility to improve co-registration accuracy would be to apply a transformation to the histology image to match the OCT image in post-processing [40]. This may help to further improve understanding of the appearance of different prostate structures in the OCT images. Using this approach, it would be necessary to carefully consider that the process of sample shrinkage during histological imaging is non-linear and is different for each type of tissues (e.g. adipose, glandular, neoplastic). However, it should be possible to account for in-plane tissue distortion using non-rigid co-registration methods. Whilst out-of-plane tissue distortions are harder to account for, previous studies have also demonstrated that this is possible [41].

The capability of OCT to perform real-time prostate imaging may eventually be translated to intraoperative tissue characterization. For example, it is often important to distinguish between healthy and cancer-affected glands intraoperatively. Our results show that generally, healthy prostate glands appear as well-defined darker areas surrounded by a lighter rim. This envelope can take on various shapes, from a clearly jagged, irregular pattern to an almost smooth pattern corresponding to naturally occurring atrophic changes, which is clearly visible in the OCT images (Fig. 5). On the other hand, the areas affected by cancer appear as more homogeneous (Fig. 6). Depending on the Gleason grade of prostate cancer determined based on histology images, brighter fragments corresponding to fibrous tissue are visible between individual cancer glands. A general trend is that with increasing Gleason score, the area visible on OCT images becomes more homogeneous (Fig. 6). Whilst this is a preliminary result, this relationship could potentially be used to distinguish different cancer grades intraoperatively. To achieve this goal, further studies are needed, including imaging of a larger number of prostate biopsies, in order to enable quantification of the obtained results. In order to obtain a more detailed differentiation between healthy and neoplastic glands with different Gleason indices, it may also be valuable to obtain additional information on the biomechanical properties of the tissues, which will be achieved as a result of parallel studies using the OCE technique.

Our study indicates that whilst OCT provides contrast of many micro-structures within the prostate, in some cases, this contrast is low. For example, nerves can be difficult to distinguish from surrounding tissues and, also, micro-structures within the nerves, such as fascicles, often present with relatively subtle variations in OCT intensity compared to surrounding structures. Also, OCT provides high contrast between glands and surrounding tissues, but in some cases, we saw evidence that glands filled with prostatic fluid can be difficult to identify, as the fluid acts to match the refractive index between glands and adjacent tissue. Functional extensions of OCT, including, OCT attenuation imaging [42], polarization-sensitive OCT [43], Doppler OCT [44], and OCE [30] have been proposed to enhance contrast in the prostate and warrant further investigation.

Currently, a key surgical focus is developing methods to improve patient outcomes in nerve-sparing radical prostatectomy [45,46]. However, there are no techniques available to provide surgeons with real-time contrast between healthy and cancerous tissues, whilst simultaneously identifying nerves. In our OCT images, nerves, especially in cross-section, are distinguishable as a darker area separated by an envelope with greater heterogeneity than the adjacent tissues that correspond to the epineurium (Fig. 3). The maximum diameter of myelinated human axons typically does not exceed 10 µm [47,48] and is below the 13 µm of OCT resolution used in this study. Consequently, it is challenging to clearly distinguish between axons and Schwann cell nuclei. However, it seems that visible inhomogeneities may correspond to individual fascicles [49]. The main goal of nerve-sparing prostatectomy is to preserve the continuity of the cavernous nerve that regulates erectile function. The average thickness of this nerve is several hundred micrometers [50] and the diameter of particular periprostatic nerve fibers ranges from 200–400 µm and, in some cases, the diameter of these nerve fibers is only several micrometers [51]. As such, the resolution of our OCT system is sufficient to see many important features of the neurovascular bundle, including the epineurium. In addition, we could adapt our method to improve its suitability for use in nerve-sparing prostatectomy, where the key requirement is the identification of the outer border of the neurovascular bundle. However, in many cases, several other important nerve features are below our OCT resolution. Whilst it was outside the scope of our study, high-resolution OCM could be used to resolve these smaller features, although it may be challenging to develop OCM for intraoperative use [52].

## 5. Conclusion

In conclusion, we have presented a feasibility study of 3-D wide-field OCT imaging of fresh, human prostate tissue. In total, we imaged 18 freshly excised prostate cross-sections and validated wide-field *en face* OCT through co-registration with wide-field histology. We demonstrated that OCT can visualize many of the important micro-structures in the prostate, including nerves, blood vessels, and glands, and that OCT can identify regions of cancer. In addition, we presented preliminary evidence that OCT contrast changes as cancer advances. We also identified some challenges in visualizing micro-architecture in the prostate. For example, nerves often present subtle contrast to surrounding tissues, and fluid-filled glands can present with low OCT contrast. We believe that this study can act as a benchmark against which to assess image quality both with compact imaging probes and functional extensions of OCT.

## Supplemental information

Visualization 1 Sequence of en face OCT images acquired at different scanning depths. https://doi.org/10.6084/m9.figshare.27169896

## Data Availability

Data underlying the results presented in this paper are not publicly available at this time but may be obtained from the authors upon reasonable request.

## References

[1] “Global cancer burden growing, amidst mounting need for services,” https://www.who.int/news/item/01-02-2024-global-cancer-burden-growing–amidst-mounting-need-for-services. PMC11115397 38438207

[2] Schlemmer H.-P. Krause B. J. Schütz V. et al., “Imaging of prostate cancer,” Dtsch. Ärztebl. Int. 118(42), 713 (2021). 10.3238/arztebl.m2021.0309 34427180 PMC8767150

[3] Turkbey B. Pinto P. A. Choyke P. L., “Imaging techniques for prostate cancer: implications for focal therapy,” Nat. Rev. Urol. 6(4), 191–203 (2009). 10.1038/nrurol.2009.27 19352394 PMC3520096

[4] Evangelista L. Zattoni F. Cassarino G. et al., “PET/MRI in prostate cancer: a systematic review and meta-analysis,” Eur. J. Nucl. Med. Mol. Imaging 48(3), 859–873 (2021). 10.1007/s00259-020-05025-0 32901351 PMC8036222

[5] Serefoglu E. C. Altinova S. Ugras N. S. et al., “How reliable is 12-core prostate biopsy procedure in the detection of prostate cancer?” Can. Urol. Assoc. J. J. Assoc. Urol. Can. 7(5-6), 293 (2013). 10.5489/cuaj.1248 PMC3668408 22398204

[6] Jiang N. Wu C. Zhou X. et al., “Cavernous nerve injury resulted erectile dysfunction and regeneration,” J. Immunol. Res. 2021, 1–10 (2021). 10.1155/2021/5353785 PMC8714392 34970630

[7] Jeong H. Choo M. S. Cho M. C. et al., “Prediction of surgical margin status and location after radical prostatectomy using positive biopsy sites on 12-core standard prostate biopsy,” Sci. Rep. 12(1), 4066 (2022). 10.1038/s41598-022-08022-5 35260742 PMC8904446

[8] Herlemann A. Cowan J. E. Carroll P. R. et al., “Community-based outcomes of open versus robot-assisted radical prostatectomy,” Eur. Urol. 73(2), 215–223 (2018). 10.1016/j.eururo.2017.04.027 28499617

[9] Oraevsky A. Ermilov S. Mehta K. et al., “In vivo testing of laser optoacoustic system for image-guided biopsy of prostate,” in Photons Plus Ultrasound: Imaging and Sensing 2006: The Seventh Conference on Biomedical Thermoacoustics, Optoacoustics, and Acousto-Optics (SPIE, 2006), 6086, pp. 80–90.

[10] Andreev V. G. Ponomarev A. E. Henrichs P. M. et al., “Detection of prostate cancer with optoacoustic tomography: feasibility and modeling,” in *Biomedical Optoacoustics IV* (SPIE, 2003), 4960, pp. 45–57.

[11] Haroon M. Tahir M. Nawaz H. et al., “Surface-enhanced Raman scattering (SERS) spectroscopy for prostate cancer diagnosis: a review,” Photodiagnosis Photodyn. Ther. 37, 102690 (2022). 10.1016/j.pdpdt.2021.102690 34921990

[12] Aubertin K. Trinh V. Q. Jermyn M. et al., “Mesoscopic characterization of prostate cancer using Raman spectroscopy: potential for diagnostics and therapeutics,” BJU Int. 122(2), 326–336 (2018). 10.1111/bju.14199 29542855

[13] Rocco B. Sighinolfi M. C. Sandri M. et al., “Digital biopsy with fluorescence confocal microscope for effective real-time diagnosis of prostate cancer: a prospective, comparative study,” Eur. Urol. Oncol. 4(5), 784–791 (2021). 10.1016/j.euo.2020.08.009 32952095

[14] Puliatti S. Bertoni L. Pirola G. M. et al., “Ex vivo fluorescence confocal microscopy: the first application for real-time pathological examination of prostatic tissue,” BJU Int. 124(3), 469–476 (2019). 10.1111/bju.14754 30908852

[15] Poola P. K. Afzal M. I. Yoo Y. et al., “Light sheet microscopy for histopathology applications,” Biomed. Eng. Lett. 9(3), 279–291 (2019). 10.1007/s13534-019-00122-y 31456889 PMC6694372

[16] Mo G. Reder N. P. Schweizer M. T., “Evaluation of initial prostate cancer biopsies utilizing 3D open-top light-sheet microscopy for detection of early disease,” The Prostate 83(11), 1121–1124 (2023). 10.1002/pros.24555 37165548

[17] Lopater J. Colin P. Beuvon F. et al., “Real-time cancer diagnosis during prostate biopsy: ex vivo evaluation of full-field optical coherence tomography (FFOCT) imaging on biopsy cores,” World J. Urol. 34(2), 237–243 (2016). 10.1007/s00345-015-1620-6 26100944

[18] Kharchenko S. Adamowicz J. Wojtkowski M. et al., “Optical coherence tomography diagnostics for onco-urology: review of clinical perspectives,” Cent. Eur. J. Urol. 66(2), 136–141 (2013). 10.5173/ceju.2013.02.art6 PMC3936153 24579012

[19] Swaan A. Mannaerts C. K. Scheltema M. J. V. et al., “Confocal laser endomicroscopy and optical coherence tomography for the diagnosis of prostate cancer: a needle-based, in vivo feasibility study protocol (IDEAL Phase 2A),” JMIR Res. Protoc. 7(5), e9813 (2018). 10.2196/resprot.9813 PMC5987046 29784633

[20] Aron M. Kaouk J. H. Hegarty N. J. et al., “Second prize: preliminary experience with the Niris TM optical coherence tomography system during laparoscopic and robotic prostatectomy,” J. Endourol. 21(8), 814–818 (2007). 10.1089/end.2006.9938 17867934

[21] D’Amico A. V. Weinstein M. Li X. et al., “Optical coherence tomography as a method for identifying benign and malignant microscopic structures in the prostate gland,” Urology 55(5), 783–787 (2000). 10.1016/S0090-4295(00)00475-1 10792101

[22] Beuvon F. Dalimier E. Cornud F. et al., “Tomographie par cohérence optique plein champ des biopsies de la prostate : un pas vers le diagnostic pré-histologique ?” Prog. En Urol. 24(1), 22–30 (2014). 10.1016/j.purol.2013.05.008 24365625

[23] Swaan A. Muller B. G. Wilk L. S. et al., “One-to-one registration of en-face optical coherence tomography attenuation coefficients with histology of a prostatectomy specimen,” J. Biophotonics 12(4), e201800274 (2019). 10.1002/jbio.201800274 30565879

[24] Hsiung P.-L. Dvm P. R. N. Fujimoto J. G., “Effect of tissue preservation on imaging using ultrahigh resolution optical coherence tomography,” J. Biomed. Opt. 10(6), 064033 (2005). 10.1117/1.2147155 16409098

[25] Gardecki J. A. Singh K. Wu C.-L. et al., “Imaging the human prostate gland using 1-µm-resolution optical coherence tomography,” Arch. Pathol. Lab. Med. 143(3), 314–318 (2019). 10.5858/arpa.2018-0135-OA 30550349

[26] Muller B. G. van Kollenburg R. A. A. Swaan A. et al., “Needle-based optical coherence tomography for the detection of prostate cancer: a visual and quantitative analysis in 20 patients,” J. Biomed. Opt. 23(08), 1–11 (2018). 10.1117/1.JBO.23.8.086001 30094972

[27] Swaan A. Mannaerts C. K. Muller B. G. et al., “The first in vivo needle-based optical coherence tomography in human prostate: a safety and feasibility study,” Lasers Surg. Med. 51(5), 390–398 (2019). 10.1002/lsm.23093 31090088 PMC6617991

[28] Rais-Bahrami S. Levinson A. W. Fried N. M. et al., “Optical coherence tomography of cavernous nerves: a step toward real-time intraoperative imaging during nerve-sparing radical prostatectomy,” Urology 72(1), 198–204 (2008). 10.1016/j.urology.2007.11.084 18280549

[29] Allen W. M. Kennedy K. M. Fang Q. et al., “Wide-field quantitative micro-elastography of human breast tissue,” Biomed. Opt. Express 9(3), 1082–1096 (2018). 10.1364/BOE.9.001082 29541505 PMC5846515

[30] Kennedy K. M. Chin L. McLaughlin R. A. et al., “Quantitative micro-elastography: imaging of tissue elasticity using compression optical coherence elastography,” Sci. Rep. 5(1), 15538 (2015). 10.1038/srep15538 26503225 PMC4622092

[31] Gibson E. Gaed M. Gómez J. A. et al., “3D prostate histology image reconstruction: Quantifying the impact of tissue deformation and histology section location,” J. Pathol. Inform. 4(1), 31 (2013). 10.4103/2153-3539.120874 24392245 PMC3869958

[32] Toren P. Venkateswaran V., “Periprostatic adipose tissue and prostate cancer progression: new insights into the tumor microenvironment,” Clin. Genitourin. Cancer 12(1), 21–26 (2014). 10.1016/j.clgc.2013.07.013 24269373

[33] Ribeiro R. Monteiro C. Cunha V. et al., “Human periprostatic adipose tissue promotes prostate cancer aggressiveness in vitro,” J. Exp. Clin. Cancer Res. 31(1), 32 (2012). 10.1186/1756-9966-31-32 22469146 PMC3379940

[34] Kennedy K. M. Zilkens R. Allen W. M. et al., “Diagnostic accuracy of quantitative micro-elastography for margin assessment in breast-conserving surgery,” Cancer Res. 80(8), 1773–1783 (2020). 10.1158/0008-5472.CAN-19-1240 32295783

[35] Kyriazis I. Spinos T. Tsaturyan A. et al., “Different nerve-sparing techniques during radical prostatectomy and their impact on functional outcomes,” Cancers 14(7), 1601 (2022). 10.3390/cancers14071601 35406373 PMC8996922

[36] Kumar A. Tandon S. Samavedi S. et al., “Current status of various neurovascular bundle-sparing techniques in robot-assisted radical prostatectomy,” J. Robot. Surg. 10(3), 187–200 (2016). 10.1007/s11701-016-0607-7 27251473

[37] Félétou M., “Introduction,” in *The Endothelium: Part 1: Multiple Functions of the Endothelial Cells—Focus on Endothelium-derived Vasoactive Mediators* (Morgan & Claypool Life Sciences, 2011). 21850763

[38] Ghani K. R. Grigor K. Tulloch D. N. et al., “Trends in reporting Gleason score 1991 to 2001: changes in the pathologist’s practice,” Eur. Urol. 47(2), 196–201 (2005). 10.1016/j.eururo.2004.07.029 15661414

[39] Liu Z. Liao Z. Wang D. et al., “Recent advances in soft biological tissue manipulating technologies,” Chin. J. Mech. Eng. 35(1), 89 (2022). 10.1186/s10033-022-00767-4

[40] Shao W. Banh L. Kunder C. A. et al., “ProsRegNet: a deep learning framework for registration of MRI and histopathology images of the prostate,” Med. Image Anal. 68, 101919 (2021). 10.1016/j.media.2020.101919 33385701 PMC7856244

[41] Winetraub Y. Van Vleck A. Yuan E. et al., “Noninvasive virtual biopsy using micro-registered optical coherence tomography (OCT) in human subjects,” Sci. Adv. 10(15), eadi5794 (2024). 10.1126/sciadv.adi5794 38598626 PMC11006228

[42] Muller B. G. de Bruin D. M. Brandt M. J. et al., “Prostate cancer diagnosis by optical coherence tomography: First results from a needle based optical platform for tissue sampling,” J. Biophotonics 9(5), 490–498 (2016). 10.1002/jbio.201500252 26856796

[43] Yoon Y. Jeon S. H. Park Y. H. et al., “Visualization of prostatic nerves by polarization-sensitive optical coherence tomography,” Biomed. Opt. Express 7(9), 3170–3183 (2016). 10.1364/BOE.7.003170 27699090 PMC5030002

[44] Standish B. A. Lee K. K. C. Jin X. et al., “Interstitial Doppler optical coherence tomography as a local tumor necrosis predictor in photodynamic therapy of prostatic carcinoma: an in vivo study,” Cancer Res. 68(23), 9987–9995 (2008). 10.1158/0008-5472.CAN-08-1128 19047181

[45] Tavukçu H. H. Aytac O. Atug F., “Nerve-sparing techniques and results in robot-assisted radical prostatectomy,” Investig. Clin. Urol. 57(Suppl 2), S172–S184 (2016). 10.4111/icu.2016.57.S2.S172 PMC5161020 27995221

[46] Jaulim A. Aydın A. Ebrahim F. et al., “Imaging modalities aiding nerve-sparing during radical prostatectomy,” Turk. J. Urol. 45(5), 325–330 (2019). 10.5152/tud.2019.19007 31509505 PMC6739086

[47] Ikeda M. Oka Y., “The relationship between nerve conduction velocity and fiber morphology during peripheral nerve regeneration,” Brain Behav. 2(4), 382–390 (2012). 10.1002/brb3.61 22950042 PMC3432961

[48] Assaf Y. Blumenfeld-Katzir T. Yovel Y. et al., “Axcaliber: A method for measuring axon diameter distribution from diffusion MRI,” Magn. Reson. Med. 59(6), 1347–1354 (2008). 10.1002/mrm.21577 18506799 PMC4667732

[49] Carolus A. Braun V. Brenke C. et al., “P 71 Optical coherence tomography – a future imaging technique of peripheral nerves?” Clin. Neurophysiol. 137, e55–e56 (2022). 10.1016/j.clinph.2022.01.102

[50] Kang J. Le H. N. D. Karakus S. et al., “Real-time, functional intra-operative localization of rat cavernous nerve network using near-infrared cyanine voltage-sensitive dye imaging,” Sci. Rep. 10(1), 6618 (2020). 10.1038/s41598-020-63588-2 32313132 PMC7171155

[51] Fried N. M. Burnett A. L., “Novel methods for mapping the cavernous nerves during radical prostatectomy,” Nat. Rev. Urol. 12(8), 451–460 (2015). 10.1038/nrurol.2015.174 26256860

[52] Nishimiya K. Tearney G., “Micro optical coherence tomography for coronary imaging,” Front. Cardiovasc. Med. 8, 613400 (2021). 10.3389/fcvm.2021.613400 33842560 PMC8032864

