# Imaging of prostate micro-architecture using three-dimensional wide-field optical coherence tomography: publisher’s note

**DOI:** 10.1364/BOE.614202

**Published:** 2026-08-18

**Authors:** Marta K. Skrok, Szymon Tamborski, Matt S. Hepburn, Qi Fang, Mateusz Maniewski, Marek Zdrenka, Maciej Szkulmowski, Adam Kowalewski, Łukasz Szylberg, Brendan F. Kennedy

**Affiliations:** 1 Institute of Physics, Faculty of Physics, Astronomy and Informatics, Nicolaus Copernicus University in Toruń, 5 Grudziądzka St., 87-100 Toruń, Poland; 2 Department of Electrical, Electronic and Computer Engineering, School of Engineering, The University of Western Australia, 35 Stirling Highway, Perth, 6009, Western Australia, Australia; 3 BRITElab, Harry Perkins Institute of Medical Research, QEII Medical Centre Nedlands and Centre for Medical Research, The University of Western Australia, Perth, Western Australia 6009, Australia; 4 Department of Obstetrics, Gynaecology and Oncology, Chair of Pathomorphology and Clinical Placentology, Collegium Medicum Jan Biziel University Hospital, 75 Ujejskiego St., Bydgoszcz 85-168, Poland; 5 Department of Tumor Pathology and Pathomorphology, Oncology Centre, Prof Franciszek Łukaszczyk Memorial Hospital, 2 Romanowskiej St., Bydgoszcz 85-796, Poland; 6 Center of Medical Sciences, University of Science and Technology, 7 Kaliskiego St., Bydgoszcz 85-796, Poland

## Abstract

This publisher’s note contains a correction to the funding of [ Biomed. Opt. Express 15, 6816 (2024)10.1364/BOE.537783PMC1164056439679405]. The article was corrected on 10 August 2026.

In [1], the funding section was amended to include award numbers.
